# ABA is required for the accumulation of APX1 and MBF1c during a combination of water deficit and heat stress

**DOI:** 10.1093/jxb/erw299

**Published:** 2016-08-06

**Authors:** Sara I. Zandalinas, Damián Balfagón, Vicent Arbona, Aurelio Gómez-Cadenas, Madhuri A. Inupakutika, Ron Mittler

**Affiliations:** ^1^Departamento de Ciencias Agrarias y del Medio Natural, Universitat Jaume I, Campus Riu Sec, E- 12071 Castello de la Plana, Spain; ^2^Department of Biological Sciences, College of Arts and Sciences, University of North Texas, 1155 Union Circle #305220, Denton, TX 76203-5017, USA

**Keywords:** *aba1-1*, *abi1-1*, abiotic stress, abscisic acid, acclimation, APX1, heat stress, MBF1c, stomata, stress combination, water deficit.

## Abstract

Abscisic acid (ABA) plays a key role in plant acclimation to abiotic stress. Although recent studies suggested that ABA could also be important for plant acclimation to a combination of abiotic stresses, its role in this response is currently unknown. Here we studied the response of mutants impaired in ABA signalling (*abi1-1*) and biosynthesis (*aba1-1*) to a combination of water deficit and heat stress. Both mutants displayed reduced growth, biomass, and survival when subjected to stress combination. Focusing on *abi1-1*, we found that although its stomata had an impaired response to water deficit, remaining significantly more open than wild type, its stomatal aperture was surprisingly reduced when subjected to the stress combination. Stomatal closure during stress combination in *abi1-1* was accompanied by higher levels of H_2_O_2_ in leaves, suggesting that H_2_O_2_ might play a role in this response. In contrast to the almost wild-type stomatal closure phenotype of *abi1-1* during stress combination, the accumulation of ascorbate peroxidase 1 and multiprotein bridging factor 1c proteins, required for acclimation to a combination of water deficit and heat stress, was significantly reduced in *abi1-1*. Our findings reveal a key function for ABA in regulating the accumulation of essential proteins during a combination of water deficit and heat stress.

## Highlight

ABA is required for plant acclimation to a combination of water deficit and heat stress regulating the accumulation of the key acclimation proteins APX1 and MBF1c.

## Introduction

Under natural conditions, or when grown in the field, plants are subjected to a combination of different abiotic stresses (Mittler, 2006; Mittler and Blumwald, 2010; Suzuki *et al.*, 2014). Recent studies identified specific physiological and molecular responses of plants to a combination of different abiotic stresses, demonstrating the importance of studying stress combination (Mittler and Blumwald, 2010; Suzuki *et al.*, 2014; Boeck *et al.*, 2015; Hu *et al.*, 2015; Liu *et al.*, 2015; Zhang *et al.*, 2015). Water deficit with high temperature represents one of the most frequent abiotic stress combinations occurring under natural conditions (Savin and Nicolas, 1996; Jiang and Huang, 2001; Mittler, 2006; Craufurd *et al.*, 2008; Boeck *et al.*, 2015). Previous studies have shown that the transcriptome of plants subjected to a combination of water deficit and heat stress is different from that of plants subjected to water deficit or heat stress alone (Rizhsky *et al.*, 2002, 2004; Mittler, 2006), suggesting that the development of broad-spectrum abiotic stress-tolerant crops will require a more detailed study of the impact of multiple environmental conditions on plants and crops (Mittler and Blumwald, 2010).

Several different phytohormones play a pivotal role in the response of plants to abiotic stress (Peleg and Blumwald, 2011; De Ollas *et al.*, 2013; Miura and Tada, 2014; Yoshida *et al.*, 2014). Abscisic acid (ABA), for example, plays a key role in the response of plants to water deficit, salinity, and heat by regulating stomatal closure and the expression of different acclimation proteins (Bartels and Sunkar, 2005; Finkelstein, 2013). Guard cell ABA signalling has been extensively studied using the ABA-insensitive dominant mutant allele *abi1-1*, which severely reduces the catalytic activity of the ABI1 type 2C protein phosphatase (Koornneef *et al.*, 1984; Merlot *et al.*, 2001). Characterization of the redox sensitivity of ABI1 revealed strong enzymatic inactivation by H_2_O_2_ (Meinhard and Grill, 2001). Production of reactive oxygen species (ROS) by respiratory burst oxidase homologue proteins, activation of Ca^2+^ channels at the plasma membrane, and activation of SLAC1, required to drive stomatal closure, were all found to be impaired in the *abi1-1* mutant (Wu *et al.*, 2003; Nilson and Assmann, 2006). Jasmonic acid (JA) is also involved in stomatal responses during abiotic stresses (Munemasa *et al.*, 2011; Daszkowska-Golec and Szarejko, 2013; Murata *et al.*, 2015), and ROS production in guard cells is also dependent on jasmonates, which interact with the ABA pathway by increasing the influx of Ca^2+^ (Munemasa *et al.*, 2011; Daszkowska-Golec and Szarejko, 2013). It has been reported that the stomata of the *abi1-1* mutant are insensitive to jasmonates and that jasmonates might affect regulation of the ABA receptor complexes in guard cells (Murata *et al.*, 2015). In addition to JA, salicylic acid (SA) can also induce stomatal closure, which is accompanied by extracellular and intracellular ROS accumulation and inward-rectifying K^+^ channel inactivation in guard cells (Khokon *et al.*, 2011).

Aside from regulating stomatal responses, ABA is involved in transcriptional regulation, for example via the ABA-responsive element (ABRE) or dehydration-responsive element (DRE; Shinozaki and Yamaguchi-Shinozaki, 2007; Umezawa *et al.*, 2010; Nakashima and Yamaguchi-Shinozaki, 2013). A recent genome‐wide search for ABRE and DRE cis‐motifs in *Arabidopsis thaliana* identified 2052 genes containing these elements (Mishra *et al.*, 2014). In addition, about 1354 genes had impaired transcript accumulation in the *abi1-1* mutant (Hoth *et al.*, 2002).

Mutants impaired in ABA signalling (*abi1-1*) or ABA biosynthesis (*aba1-1*) were recently reported to be impaired in their acclimation to a combination of salt and heat stress (Suzuki *et al.*, 2016), suggesting an important role for ABA in plant acclimation to abiotic stress combination. Nevertheless, it is not known whether this impairment is due to ABA’s role in regulating stomatal responses, transcript expression, or both (Suzuki *et al.*, 2016).

Here we report that *abi1-1* and *aba1-1* plants are impaired in their acclimation to a combination of water deficit and heat stress. Focusing on the *abi1-1* mutant, we found that although its stomata displayed an impaired response to water deficit stress, remaining significantly more open than wild type, its stomatal aperture was surprisingly reduced when plants were subjected to a combination of water deficit and heat stress, similar to wild type. This demonstrates a potentially unique stomatal regulation mechanism during stress combination. Stomatal closure in the *abi1-1* mutant during stress combination was accompanied by higher levels of H_2_O_2_ in leaves, suggesting that H_2_O_2_ might play a role in this response. In contrast to the almost wild-type response of stomatal closure displayed by *abi1-1* plants during the stress combination, the accumulation of ascorbate peroxidase 1 (APX1) and multiprotein bridging factor 1c (MBF1c), two proteins required for plant survival during a combination of water deficit and heat stress (Suzuki *et al.*, 2005; Koussevitzky *et al.*, 2008), was significantly reduced in the *abi1-1* mutant compared to wild type. Our findings reveal a potential role for H_2_O_2_ in regulating stomatal responses during stress combination and point to a key role for ABA in regulating the accumulation of APX1 and MBF1c during a combination of water deficit and heat stress.

## Materials and methods

### Plant material, growth conditions, and stress treatments

*Arabidopsis thaliana* L*er* (cv Landsberg erecta), *aba1-1*, and *abi1-1* (Assmann *et al.*, 2000) plants were grown in 240-cm^2^ inserts on soil mixture (MetroMix 200, SUN GRO) under controlled conditions: 21°C, 10-h light cycle, 100 μmol m^−2^ s^−1^, and relative humidity of 70% (AR-66, Percival Scientific) as described in Suzuki *et al.* (2016). All stress treatments were performed in parallel as described in Rizhsky *et al.* (2004) with the following modifications: A water deficit was applied by withdrawing water from 10-day-old plants until reaching 40% of control soil weight, typically within 20–25 days. Heat stress was imposed by transferring 30-day-old plants to 38°C for 8h as follows: 06:00–08:00, 21°C; 08:00–16:00, 38°C. The water deficit and heat stress combination was performed by applying heat stress to 30-day-old plants under water deficit (Supplementary Fig. S1). Rosettes from L*er* and *abi1-1* plants were sampled at the same time and all measurements were performed in parallel after each stress condition (Supplementary Fig. S1). Following the stress treatments, plants were recovered under controlled conditions for 5 days and scored for survival. Temperature and relative humidity were recorded regularly with a portable USB datalogger (OM-EL-USB-2-LCD-PLUS, OMEGA Engineering, Inc., Stamford, CT, USA; Supplementary Fig. S1B). All experiments were repeated at least three times.

### Growth characteristics

Fresh weight (FW), dry weight (DW), and plant diameter were measured as described in Suzuki *et al.*, (2005, 2016). Relative water content (RWC) was measured using rosettes, which were immediately weighed after stress treatments to obtain FW. Rosettes were then placed in a beaker of water overnight in the dark, allowing them to become fully hydrated. They were then reweighed to obtain turgid weight (TW) and dried at 40ºC for 4 days to obtain DW. Finally, RWC (%) was calculated as [(FW − DW) × (TW − DW)^−1^] × 100. For plant survival, plants were recovered for 5 days under controlled conditions (well-watered and 21°C) and the percentage survival of plants was scored and calculated as described in Koussevitzky *et al.* (2008) and Suzuki *et al.* (2016).

### Stomatal conductance

Stomatal conductance (gs) was measured in parallel on *aba1-1* and *abi1-1* plants of each treatment using an LCpro+ portable infrared gas analyser (ADC BioScientific Ltd., Hoddesdon, UK). After instrument stabilization, at least 10 measurements were taken on three leaves in three replicate plants from each mutant and stress treatment.

### Plant hormone analysis

Hormone extraction and analysis were carried out as described in Durgbanshi *et al.* (2005) with few modifications. Briefly, 0.1g of dry tissue was extracted in 2mL of ultrapure water after spiking with 50ng of [^2^H_6_]-ABA, [C_13_]-SA, and dihydrojasmonic acid in a ball mill (MillMix20, Domel, Železniki, Slovenija). After centrifugation at 4000 g at 4ºC for 10min, supernatants were recovered and pH adjusted to 3 with 30% acetic acid. The water extract was partitioned twice against 2mL of diethyl ether and the organic layer recovered and evaporated under vacuum in a centrifuge concentrator (Speed Vac, Jouan, Saint Herblain Cedex, France). Once dried, the residue was resuspended in a 10:90 MeOH:H_2_O solution by gentle sonication. The resulting solution was filtered through 0.22 µm polytetrafluoroethylene membrane syringe filters (Albet S.A., Barcelona, Spain) and directly injected into an ultra performance LC system (Acquity SDS, Waters Corp., Milford, MA, USA). Chromatographic separations were carried out on a reversed-phase C18 column (Gravity, 50×2.1mm, 1.8-µm particle size, Macherey-Nagel GmbH, Germany) using a MeOH:H_2_O (both supplemented with 0.1% acetic acid) gradient at a flow rate of 300 µL min^−1^. Hormones were quantified with a TQS triple quadrupole mass spectrometer (Micromass, Manchester, UK) connected online to the output of the column though an orthogonal Z-spray electrospray ion source.

### Stomatal aperture

Stomatal aperture analysis was performed as described in Morillon and Chrispeels (2001). Briefly, three leaves of L*er* and *abi1-1* from each plant were cut and the lower surface was immediately stuck to a microspore slide with a medical adhesive (Hollister, Libertyville, IL, USA). After 1–2min, the leaf was peeled away under distilled water. The lower epidermis stuck to the glass was visualized under the microscope and stomatal images were recorded. Measurements of stomatal aperture were performed using the imaging software Image J, version 6.

### H_2_O_2_ measurement

H_2_O_2_ accumulation in rosette tissues was measured using the Amplex Red Hydrogen Peroxide-Peroxidase Assay kit (Molecular Probes, Invitrogen, Carlsbad, CA, USA) as described in Suzuki *et al.* (2015). In brief, 500 µL of 50-mM sodium phosphate buffer (pH 7.4) containing 50 µM Amplex Red and 0.05 U mL^−1^ horseradish peroxidase was added to ground, frozen tissues. Samples were centrifuged at 12 000g for 12min at 4°C. Next, 450 µL of supernatant was transferred into fresh tubes and incubated for 30min at room temperature in the dark. Absorbance at 560nm was measured using a NanoDrop spectrophotometer (Thermo Scientific, Wilmington, DE, USA). The concentration of H_2_O_2_ in each sample was determined from a standard curve consisting of 0, 0.5, 1, 3, 6, and 9 µM of H_2_O_2_. Following the measurement of absorbance, tissue samples were completely dried using a speed vacuum concentrator for 90min and H_2_O_2_ accumulation per gram of dry weight was calculated.

### H_2_O_2_ and ABA treatments

H_2_O_2_ and ABA treatments were conducted by spraying 1mM H_2_O_2_ or 30 µM ABA on 30-day-old L*er* and *abi1-1* plants. Control plants were simultaneously sprayed with distilled water. Stomatal aperture for control and treated leaves was measured after 30 and 60min of each treatment in plants kept in the light or in the dark.

### Protein blot analysis

Protein was isolated, quantified, and analysed by protein blot as previously described (Miller *et al.*, 2007). Coomassie Blue staining of protein gels was used to control for protein loading.

### Statistical analysis

Genotypic differences were discriminated by a one-tailed Student’s *t*-test. Results are presented as the mean ± SD (*P* < 0.05). Stress acclimation data were subjected to a two-way ANOVA with the interaction genotype × stress followed by a Tukey post hoc test (*P* < 0.05) when a significant difference was detected (Supplementary Table S1).

## Results

### Growth of wild-type (L*er*), *aba1-1*, and *abi1-1* plants subjected to a combination of water deficit and heat stress

**Fig. 1.**
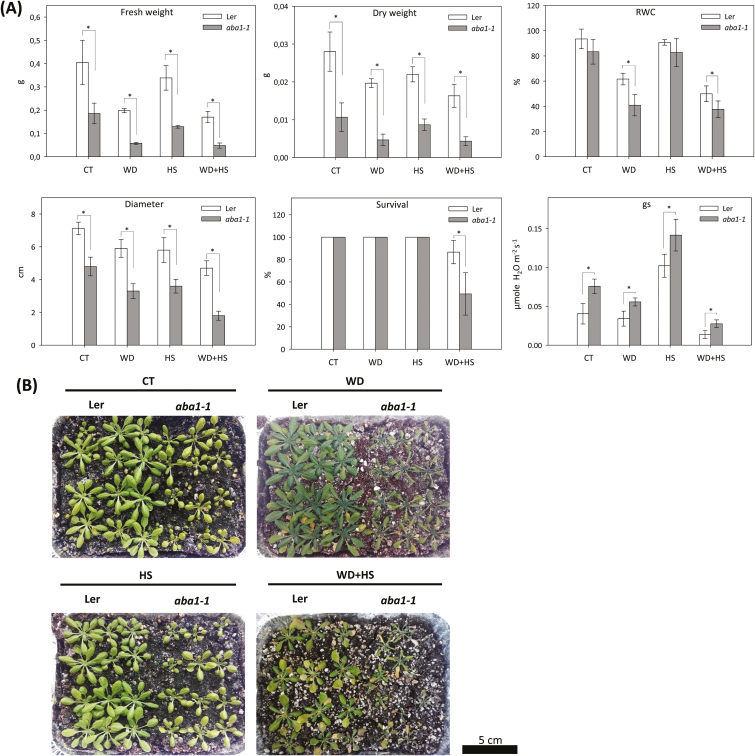
Growth, biomass, survival, RWC, and stomatal conductance of wild-type and *aba1-1* plants subjected to a combination of water deficit and heat stress. (A) Shoot fresh and dry weight (g; average of five individual rosettes), RWC, rosette diameter, survival, and stomatal conductance (gs) of plants subjected to water deficit (WD), heat stress (HS), and a combination of water deficit and heat stress (WD+HS). * Student’s *t*-test significant at *P* < 0.05. Error bars represent SD. (B) Representative images of wild-type and *aba1-1* plants subjected to the different stresses. CT, control.

**Fig. 2.**
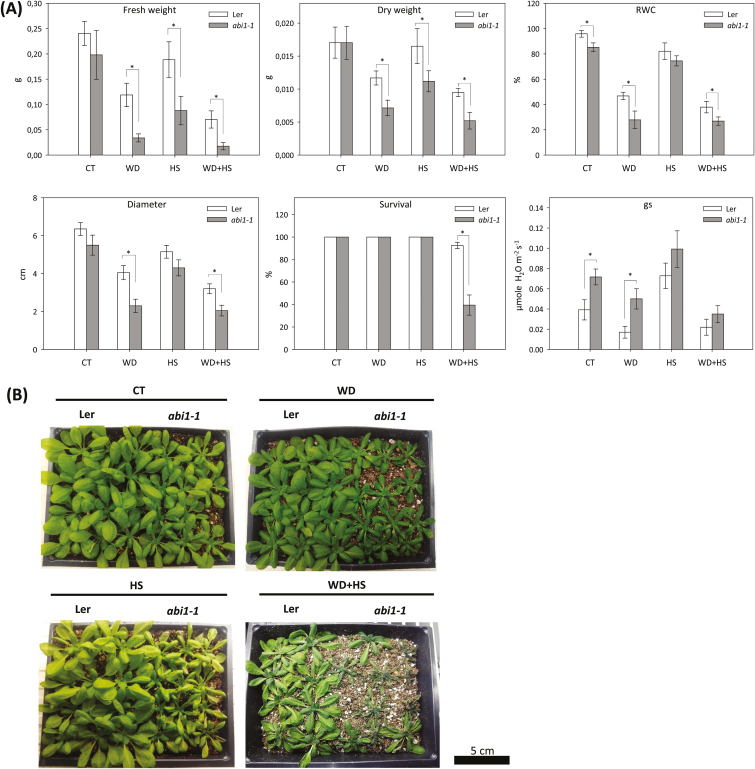
Growth, biomass, survival, RWC, and stomatal conductance of wild-type and *abi1-1* plants subjected to a combination of water deficit and heat stress. (A) Shoot fresh and dry weight (g; average of five individual rosettes), RWC, rosette diameter, survival, and stomatal conductance (gs) of plants subjected to water deficit (WD), heat stress (HS), and a combination of water deficit and heat stress (WD+HS). * Student’s *t*-test significant at *P* < 0.05. Error bars represent SD. (B) Representative images of wild-type and *abi1-1* plants subjected to the different stresses. CT, control.

Rosette FW and DW, RWC, plant diameter, survival and gs of L*er*, *aba1-1*, and *abi1-1* plants subjected to water deficit, heat stress, and a combination of water deficit and heat stress were characterized (Figs 1, 2). Compared to wild-type L*er* plants, *aba1-1* plants showed a decrease in FW, DW, and rosette diameter in response to all stress treatments. In addition, water deficit and a combination of water deficit and heat stress significantly reduced RWC, whereas gs increased in *aba1-1* in response to all stress treatments. Plant survival under combined stress conditions was 90% in wild-type plants and 50% in *aba1-1* plants (Fig. 1A). Compared to wild type, *abi1-1* plants showed a significant reduction in FW and DW in response to all stress treatments. In contrast, a decreased diameter and RWC were only observed in *abi1-1* plants in response to water deficit or water deficit combined with heat stress (Fig. 2A). Whereas 95% of wild-type plants survived the stress combination, only about 40% of *abi1-1* survived exposure to a combination of water deficit and heat stress (Fig. 2A). Increased gs was observed in *abi1-1* plants compared to wild-type plants in control conditions as well as in response to water deficit (Fig. 2A). The results shown in Figs 1 and 2 demonstrate that although the growth of *abi1-1* and *aba1-1* plants was negatively impacted by water deficit or heat stress compared to wild type, these treatments had no adverse effect on survival. In contrast, the combination of water deficit and heat stress significantly impacted the survival of *abi1-1* and *aba1-1* plants, demonstrating that mutants impaired in ABA biosynthesis or signalling are impaired in their acclimation to this stress combination. A *genotype × stress* interaction analysis further confirmed the dependency of plant survival on ABA function with a *P* value ≤ 0.001 (Supplementary Table S1).

To further study the role of ABA in plant acclimation to a combination of water deficit and heat stress, we focused our studies on the *abi1-1* mutant that is impaired in ABA signalling (Assmann *et al.*, 2000; Wu *et al.*, 2003; Nilson and Assmann, 2006).

### Stomatal aperture of L*er* and *abi1-1* plants subjected to a combination of water deficit and heat stress

**Fig. 3.**
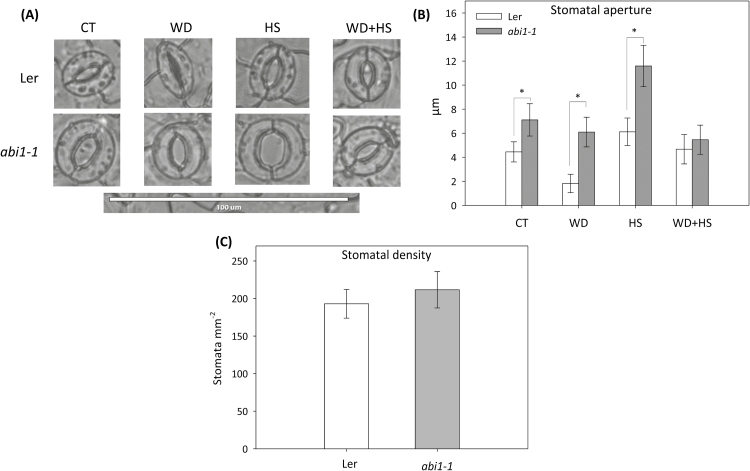
Stomatal aperture of wild-type and *abi1-1* plants subjected to a combination of water deficit and heat stress. (A) Representative images of stomata from wild-type and *abi1-1* plants subjected to water deficit (WD), heat stress (HS), and a combination of water deficit and heat stress (WD+HS). (B) Measurements of stomatal aperture of wild-type and *abi1-1* plants subjected to the different stresses. (C) Stomatal density of wild-type and *abi1-1* plants. * Student’s *t*-test significant at *P* < 0.05. Error bars represent SD. CT, control.

To determine whether the differences observed between the survival of *abi1-1* and wild-type plants in response to the stress combination (Fig. 2) were related to the impaired stomatal responses of *abi1-1* (Wu *et al.*, 2003; Nilson and Assmann, 2006), the stomatal aperture of L*er* and *abi1-1* plants was measured under water deficit, heat stress, and a combination of water deficit and heat stress (Fig. 3A, B). Under controlled conditions, the stomatal aperture of *abi1-1* plants was larger than that of wild type (Fig. 3A, B). The stomatal aperture of *abi1-1* plants did not decrease in response to water deficit, reflecting the impairment of *abi1-1* in stomatal responses (Fig. 3A, B; Wu *et al.*, 2003; Nilson and Assmann, 2006). In contrast, the stomatal aperture of *abi1-1* increased in response to heat stress and was larger than that of wild type, showing that the stomata of *abi1-1* could open in response to heat stress (Fig. 3A, B). Surprisingly, and in contrast to the differences in stomatal responses and stomatal aperture observed between wild-type and *abi1-1* plants under controlled conditions, heat stress, or water deficit (Fig. 3A, B), in response to the stress combination the stomatal aperture of *abi1-1* was reduced to levels similar to that of wild-type plants (Fig. 3A, B). The measurements of stomatal aperture in control and *abi1-1* plants during stress combination (Fig. 3A, 3B) were in agreement with the gs measurements of control and *abi1-1* subjected to the stress combination (Fig. 2A), providing further confidence in this finding. To determine whether the density of stomata in the *abi1-1* mutant would be a factor in affecting overall transpiration during the different stresses, we also measured the stomatal density of wild-type and *abi1-1* plants. As shown in Fig. 3C, no significant difference was observed between wild-type and *abi1-1* plants, both harbouring about 200 stomata per mm^2^. The results shown in Fig. 3 could explain the differences between the reduced growth, biomass, and RWC of *abi1-1* and wild-type plants shown in Fig. 2, but likely not the differences in *abi1-1* survival in response to the stress combination (Fig. 2).

### Accumulation of ABA, JA, and SA in wild-type and *abi1-1* plants subjected to a combination of water deficit and heat stress

**Fig. 4.**
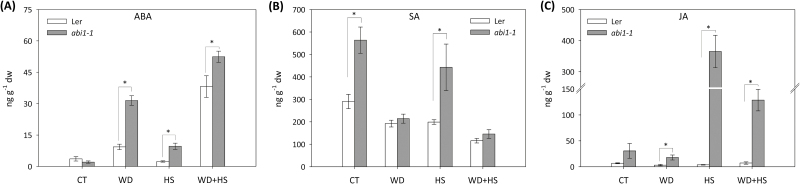
Accumulation of ABA, JA, and SA in wild-type and *abi1-1* plants subjected to a combination of water deficit and heat stress. ABA (A), SA (B), and JA (C) accumulation in wild-type and *abi1-1* plants subjected to water deficit (WD), heat stress (HS), and a combination of water deficit and heat stress (WD+HS). * Student’s *t*-test significant at *P* < 0.05. Error bars represent SD. CT, control.

To examine whether the differences in stomatal responses and plant survival observed between wild-type and *abi1-1* plants in response to the stress combination (Figs 2, 3) were related to the accumulation of ABA, JA, and/or SA in leaves, we measured the levels of these hormones in wild-type and *abi1-1* plants subjected to the different stresses (Fig. 4). As shown in Fig. 4A, water deficit and a combination of water deficit and heat stress was accompanied by an accumulation of ABA, but not SA or JA, in wild-type plants. In addition, JA and SA did not accumulate in wild-type leaves in response to water deficit, heat stress, or their combination (Fig. 4B, C). In contrast, *abi1-1* plants accumulated high levels of ABA in response to all stress treatments (Fig. 4A; this observation was further supported by correlation analysis between RWC and ABA in plants subjected to the different stresses; Supplementary Fig. S2, Supplementary Table S1), high levels of SA under control conditions and in response to heat stress (Fig. 4B), and high levels of JA in response to heat stress and a combination of water deficit and heat stress (Fig. 4C). The combination of water deficit and heat stress resulted in the highest accumulation of ABA in both *abi1-1* and wild-type plants, suggesting that ABA is important for plant acclimation to this stress combination. The results shown in Fig. 4 could implicate JA-related pathways in the stomatal aperture reduction response of *abi1-1* plants during the stress combination (Fig. 3). In addition, they reflect the deficiency in ABA signalling in *abi1-1* plants that results in higher accumulation of ABA in response to stress (Fig. 4A).

### H_2_O_2_ accumulation in wild-type and *abi1-1* plants in response to a combination of water deficit and heat stress

**Fig. 5.**
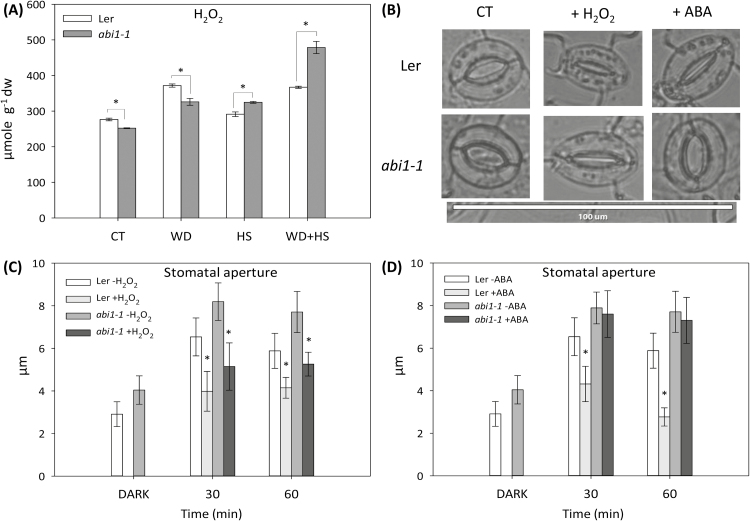
H_2_O_2_ accumulation in wild-type and *abi1-1* plants in response to a combination of water deficit and heat stress. (A) H_2_O_2_ accumulation in wild-type and *abi1-1* plants subjected to water deficit (WD), heat stress (HS), and a combination of water deficit and heat stress (WD+HS). (B) Representative images of stomata of *Arabidopsis* plants 60min after the application of H_2_O_2_ or ABA. (C) Measurements of stomatal aperture of wild-type and *abi1-1* plants following application of 1mM H_2_O_2_. (D) Measurements of stomatal aperture of wild-type and *abi1-1* plants following application of 30 µM ABA. * Student’s *t*-test significant at *P* < 0.05. Error bars represent SD. CT, control.

H_2_O_2_ plays an important role in abiotic stress and stomatal responses (Qiao *et al.*, 2014; Song *et al.*, 2014). To determine whether H_2_O_2_ plays a role in the regulation of stomatal aperture during a combination of water deficit and heat stress, we measured the levels of H_2_O_2_ in leaves from plants subjected to the different stresses. As shown in Fig. 5A, H_2_O_2_ accumulated in wild-type plants in response to water deficit and a combination of water deficit and heat stress, but not to heat stress alone. In the *abi1-1* mutant, H_2_O_2_ accumulated in response to all stress treatments, with the highest levels obtained in *abi1-1* plants subjected to a combination of water deficit and heat stress (Fig. 5A). The high levels of H_2_O_2_ measured in the leaves of the *abi1-1* mutant in response to the stress combination (Fig. 5A) could explain the closure of stomata in the *abi1-1* mutant during a combination of water deficit and heat stress (Fig. 3). We therefore examined how H_2_O_2_ or ABA application would affect the stomatal aperture of wild-type and *abi1-1* plants. As shown in Fig. 5B, D, application of H_2_O_2_ (1mM), but not ABA (30 µM), resulted in a significant reduction of stomatal aperture in the *abi1-1* mutant. The results presented in Fig. 5 could implicate H_2_O_2_ as an important signalling molecule that promotes stomatal closure in wild type and the *abi1-1* mutant during abiotic stress combination.

### Accumulation of key acclimation proteins involved in the response of plants to a combination of water deficit and heat stress in wild-type and *abi1-1* plants

**Fig. 6.**
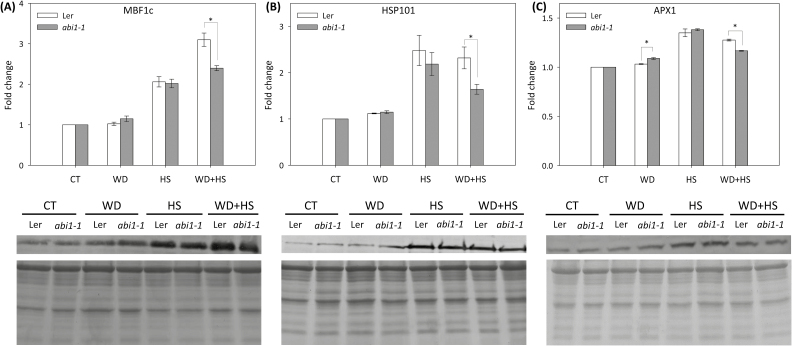
Accumulation of key acclimation proteins involved in the response of plants to a combination of water deficit and heat stress in wild-type and *abi1-1* plants. Protein blot analysis of MBF1c (A), HSP101 (B), and APX1 (C) accumulation in leaves of wild-type and *abi1-1* plants subjected to water deficit (WD), heat stress (HS), and a combination of water deficit and heat stress (WD+HS). Top: Quantification bar graphs for fold change in protein accumulation. Bottom: Protein blots and Coomassie-stained gels of total protein. Quantification of protein expression was performed per total protein for three different experiments. * Student’s *t*-test significant at *P* < 0.05. Error bars represent SD. CT, control.

Although the *abi1-1* mutant was more susceptible to a combination of water deficit and heat stress than wild type (Fig. 2), this susceptibility was not reflected in its stomatal responses to the stress combination (Fig. 3), and therefore may not be explained by excessive water loss of the *abi1-1* mutant during the stress combination. ABA plays a dual role during the response of plants to abiotic stress, controlling stomatal responses as well as stress-response transcript and protein expression. We thus measured the accumulation of three key proteins important for plant acclimation to osmotic, heat, or oxidative stress (HSP101, APX1, and MBF1c; Queitsch *et al.*, 2000; Arce *et al.*, 2010; Suzuki *et al.*, 2011), or a combination of water deficit and heat stress (MBF1c and APX1; Suzuki *et al.*, 2005; Koussevitzky *et al.*, 2008), in wild-type plants and *abi1-1* mutants subjected to water deficit, heat stress, and their combination. As shown in Fig. 6, compared to wild-type plants, the accumulation of all three proteins was suppressed in the *abi1-1* mutant in response to the stress combination. Because the expression of APX1 and MBF1c is required for plant acclimation to a combination of water deficit and heat stress (Suzuki *et al.*, 2005; Koussevitzky *et al.*, 2008), the results shown in Fig. 6 could explain the higher susceptibility of the *abi1-1* mutant to the stress combination (Fig. 2), as well as highlight the important role ABA could play in the regulation of acclimation mechanisms during abiotic stress combination.

### Meta-analysis of transcriptomics data from ABA-treated *abi1-1* and wild-type plants, and wild-type plants subjected to a combination of water deficit and heat stress

**Fig. 7.**
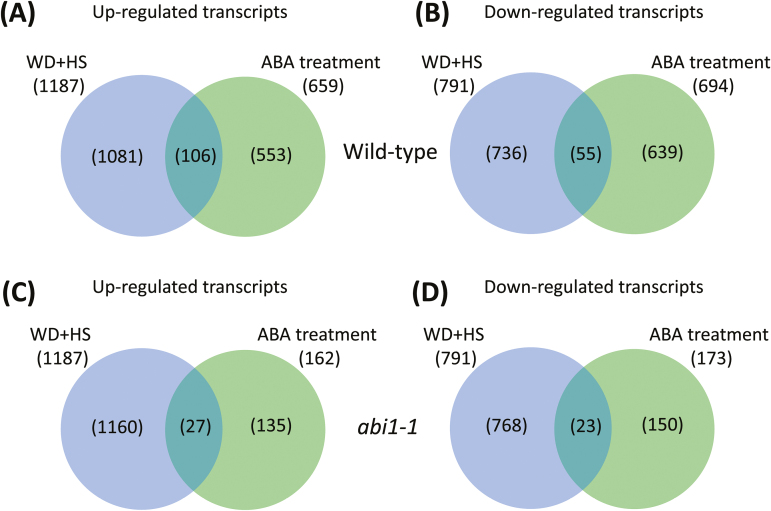
Meta-analysis of transcriptomics data from ABA-treated *abi1-1* and wild-type (L*er*) plants, and wild-type (Col) plants subjected to a combination of water deficit and heat stress. Top: Venn diagrams showing the overlap between transcripts specifically up-regulated (A) or down-regulated (B) in response to a combination of water deficit and heat stress or to ABA treatment in wild-type plants. Bottom: Venn diagrams showing the overlap between transcripts specifically up-regulated (C) or down-regulated (D) in response to a combination of water deficit and heat stress in wild-type plants and ABA treatment in *abi1-1* plants. References used for the meta-analysis are Hoth *et al.* (2002) and Rizhsky *et al.* (2004).

The results presented in Fig. 6 strongly support a role for ABA in the accumulation of different acclimation proteins during water deficit and heat stress combination. To examine how broad this role might be, we compared the transcriptome of wild-type plants (Col) subjected to a combination of water deficit and heat stress (Rizhsky *et al.*, 2004) with that of wild-type plants (L*er*) treated with 50 µM ABA (Hoth *et al.*, 2002). As shown in Fig. 7A, 106 transcripts were common between the 1187 up-regulated transcripts during water deficit and heat stress combination (Rizhsky *et al.*, 2004) and the 659 transcripts up-regulated following ABA treatment of unstressed wild-type plants (Hoth *et al.*, 2002). An overlap of 55 transcripts was found between the 791 down-regulated transcripts during water deficit and heat stress combination and the 694 transcripts down-regulated following ABA treatment of unstressed wild-type plants (Fig. 7B). When the same overlap was tested between transcripts up- or down-regulated in wild-type (Col) plants in response to a combination of water deficit and heat stress (Rizhsky *et al.*, 2004), and transcripts up- or down-regulated in the *abi1-1* mutant (L*er*) in response to ABA treatment of unstressed plants (50 µM; Hoth *et al.*, 2002) (Fig. 7C, D), it was found that the number of overlapped transcripts between the two groups decreased by about 75% (up-regulated) and 50% (down-regulated). This finding could suggest that in addition to HSP101, MBF1c, and APX1 (Fig. 6), *abi1-1* could be involved in the regulation of several other acclimation pathways in plants in response to a combination of water deficit and heat stress. A list of the transcripts that could be under the control of *abi1-1* in response to the stress combination is included in Supplementary Table S2.

## Discussion

We recently conducted transcriptome analysis of Arabidopsis plants subjected to a combination of salinity and heat stress and identified many ABA-response transcripts within the group of transcripts that were specifically expressed in response to the stress combination (Suzuki *et al.*, 2016). We subsequently determined that mutants impaired in ABA synthesis (*aba1-1*) or ABA signalling (*abi1-1*) were more susceptible than wild-type plants to a combination of salinity and heat stress (Suzuki *et al.*, 2016). Nonetheless, whether this enhanced susceptibility to the stress combination was due to impaired stomatal responses, deficiency in the expression of different acclimation transcripts and proteins, or both, was unclear.

To deepen our understanding of ABA’s role in the response of plants to a combination of different abiotic stresses, we focused in the current study on the acclimation of plants to a combination of water deficit and heat stress. Our findings that the *abi1-1* and *aba1-1* mutants are susceptible to this stress combination (Figs 1, 2; Supplementary Table S1), that the stress combination was accompanied by elevated accumulation of ABA in wild-type and *abi1-1* plants (Fig. 4), and that many transcripts involved in the response of plants to a combination of water deficit and heat stress are ABA-response transcripts (Fig. 7), demonstrate that ABA is required for the acclimation of plants to yet another type of stress combination, that is, water deficit and heat stress. These findings underscore a possible general role for ABA in the acclimation of plants to abiotic stress combinations. Further studies focused on the acclimation of mutants deficient in ABA metabolism and signalling to additional abiotic stress combinations would reveal how broad the role of ABA is in the response of plants to stress combination.

We further focused on the *abi1-1* mutant and examined whether ABA is required for stomatal responses, expression of different acclimation transcripts and proteins, or both (Figs 3, 6). Surprisingly, we found that the stomata of *abi1-1* plants, although impaired in their responses to water deficit (Figs 2, 3), reduced their aperture to levels that are similar to that of wild-type plants in response to the combination of heat stress and water deficit (Fig. 3). This finding suggested that ABI1 might not be required for stomatal closure during the stress combination, and that the decreased survival of *abi1-1* plants subjected to the stress combination (Fig. 2) can not be simply explained by enhanced water loss during the stress combination due to impaired stomatal responses (although some loss of RWC was observed in *abi1-1* plants during the stress combination; Fig. 2A). In contrast to the almost wild-type stomatal phenotype of *abi1-1* plants under the stress combination (Fig. 3), the accumulation of proteins important for plant acclimation to heat stress (HSP101 and MBF1c) and a combination of water deficit and heat stress (MBF1c and APX1) was attenuated in *abi1-1* plants during the stress combination (Fig. 6). Taken together, the findings shown in Figs 3 and 6 suggest that the cause of the enhanced susceptibility of *abi1-1* plants to the stress combination (Fig. 2) could be an outcome of the inability of these plants to mount an acclimation response involving the accumulation of MBF1c and APX1 (Fig. 6), as opposed to their inability to close their stomata (Fig. 3). ABA may therefore be required for the accumulation of key proteins required for the acclimation of plants to a combination of different stresses (Fig. 6).

**Fig. 8.**
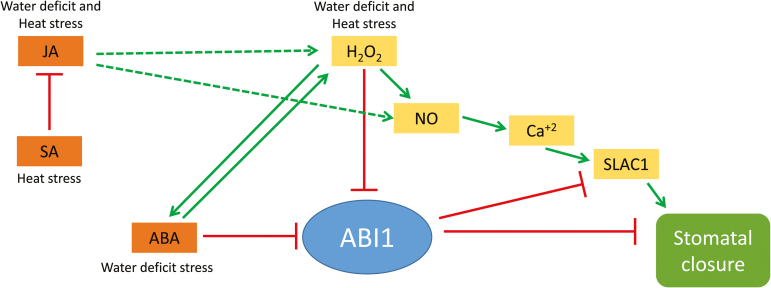
A hypothetical model for the signalling role of JA, SA, ABA, and H_2_O_2_ in the regulation of stomatal aperture in *abi1-1* during water deficit, heat stress, and a combination of water deficit and heat stress. Dotted lines indicate hypothetical interactions. Solid lines and arrows indicate positive and negative regulation based on published literature, respectively. Abbreviations: ABA, abscisic acid; JA, jasmonic acid; NO, nitric oxide; SA, salicylic acid; SLAC1, SLOW ANION CHANNEL-ASSOCIATED 1.

In an attempt to address the question of how the stomata of *abi1-1* had reduced aperture during the stress combination, we measured the levels of ABA, JA, SA, and H_2_O_2_ in plants subjected to the stress combination. As shown in Figs 4 and 5, the stress combination was accompanied by a unique combination of high H_2_O_2_, high JA, and low SA in leaves of *abi1-1* plants. Under this combination, H_2_O_2_ and JA can signal stomatal closure, independent of ABA signalling, by enhancing nitric oxide (NO) levels and triggering Ca^2+^ and SLAC1 function (Fig. 8; Daszkowska-Golec and Szarejko, 2013; Murata *et al.*, 2015). In contrast, during heat stress when both SA and JA are enhanced (Fig. 4), SA could antagonize JA function and stomata will open in *abi1-1* (Figs 3, 8; Thaler *et al.*, 2012; Caarls *et al.*, 2015). In support of the proposed role of H_2_O_2_ in mediating stomatal closer in *abi1-1* plants, the application of H_2_O_2_, but not ABA, was able to induce stomatal closer in unstressed *abi1-1* plants (Fig. 5B, D). Our findings point to an alternative pathway that may be involved in the stomatal responses of plants subjected to stress combination, involving JA and/or H_2_O_2_ (Fig. 8). Further studies are of course needed to address this possibility, including the analysis of additional mutants in ABA, JA, and ROS signalling and direct measurements of H_2_O_2_, JA, SA, NO, and ABA in the stomata of plants subjected to stress combination. Because ABA is required for different physiological acclimation responses, as well as for the regulation of protein and transcript accumulation during different stresses and their combination, ABA could function as an overall regulator that tailors the plant response to the different environmental conditions.

## Supplementary data

Supplementary data are available at *JXB* online.

Figure S1. Experimental design, and temperature and humidity measurements.

Figure S2. Correlation analysis between RWC and hormonal concentrations (ABA, SA, and JA) obtained for L*er* and *abi1-1* plants under water deficit, heat stress, and a combination of water deficit and heat stress.

Table S1. Analysis of variance of growth characteristic parameters and hormonal concentrations for *aba1-1* and *abi1-1* plants.

Table S2. List of the transcripts that could be under the control of *abi1-1* in response to the stress combination.

Supplementary Data
