# Supplementary material for: ABA is required for the accumulation of APX1 and MBF1c during a combination of water deficit and heat stress
Source: J Exp Bot. 2016 Aug 6;67(18):5381–90. doi: 10.1093/jxb/erw299 (PMC5049388; doi:10.1093/jxb/erw299)
Supplement: Supplementary Data [file supp_67_18_5381__index.html]

ABA is required for the accumulation of APX1 and MBF1c during a combination of water deficit and heat stress — ABA is required for the accumulation of APX1 and MBF1c during a combination of water deficit and heat stress — Supplementary Data 

# ABA is required for the accumulation of APX1 and MBF1c during a combination of water deficit and heat stress

## Supplementary Data

Data files

- supplementary\_figure\_S1\_S2\_table\_S1\_S2.pdf - Supplementary Data
