# Supplementary material for: ABA is required for the accumulation of APX1 and MBF1c during a combination of water deficit and heat stress
Source: J Exp Bot. 2016 Aug 6;67(18):5381–90. doi: 10.1093/jxb/erw299 (PMC5049388; doi:10.1093/jxb/erw299)
Supplement: Supplementary Data [file supp_erw299_supplementary_figure_S1_S2_table_S1_S2.pdf]

Supplementary Material

Figure S1 (A) The experimental design used to subject wild type, *aba1-1* and *abi1-1* plants to water deficit (yellow), heat stress (red) and a combination of water deficit and heat stress (orange). **(B)** Temperature and humidity measurements for control (CT), water deficit (WD), heat stress (HS) and a combination of water deficit and heat stresses (WD+HS), recorded inside the growth chamber with a datalogger.

Figure S2 Correlation analysis between RWC and hormonal concentrations (ABA, SA and JA) obtained for Ler and *abi1-1* plants under water deficit, heat stress and a combination of water deficit and heat stress.

Table S1 Analysis of variance of growth characteristic parameters and hormonal concentrations for *aba1-1* and *abi1-1* plants. $P<0.05$ denotes statistical significance.

Table S2 List of the transcripts that could be under the control of *abi1-1* in response to the stress combination.

Figure S1 (A) The experimental design used to subject wild type, *aba1-1* and *abi1-1* plants to water deficit (yellow), heat stress (red) and a combination of water deficit and heat stress (orange). **(B)** Temperature and humidity measurements for control (CT), water deficit (WD), heat stress (HS) and a combination of water deficit and heat stresses (WD+HS), recorded inside the growth chamber with a datalogger.

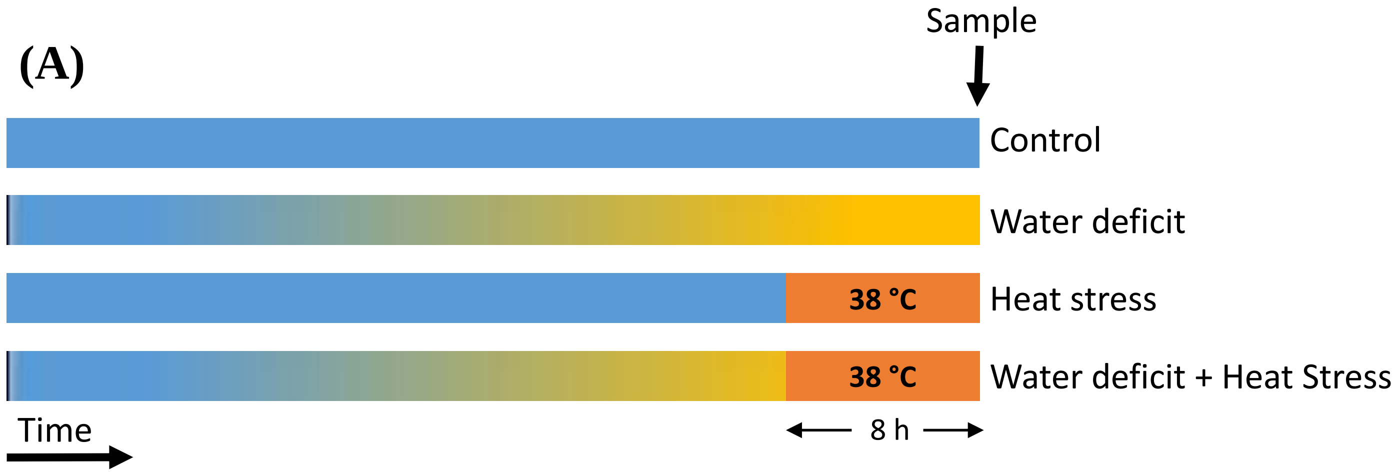

(B)

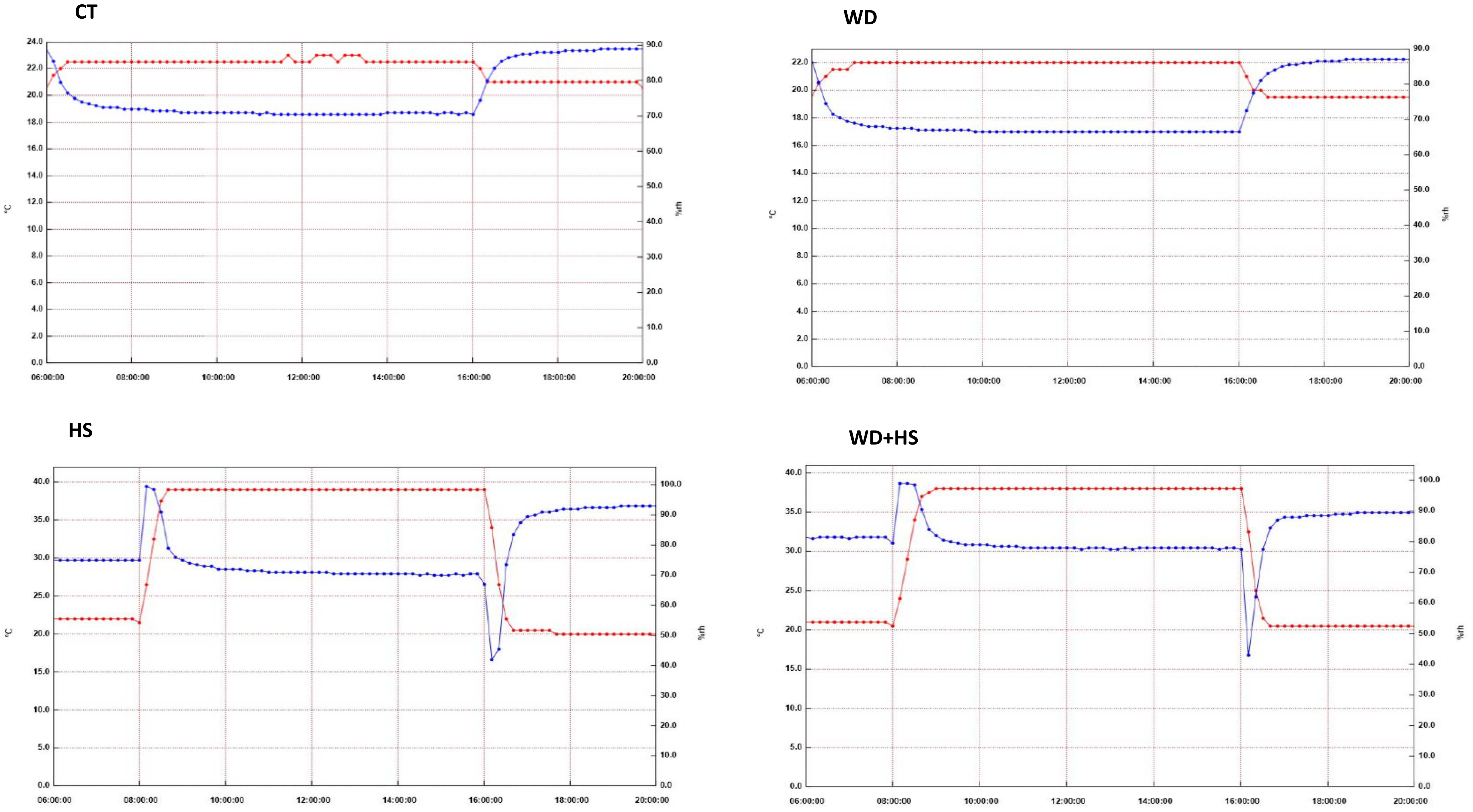

CT
WD
HS
WD+HS
°C
%rh
06:00:00
08:00:00
10:00:00
12:00:00
14:00:00
16:00:00
18:00:00
20:00:00

Figure S2 Correlation analysis between RWC and hormonal concentrations (ABA, SA and JA) obtained for Ler and *abi1-1* plants under water deficit, heat stress and a combination of water deficit and heat stress.

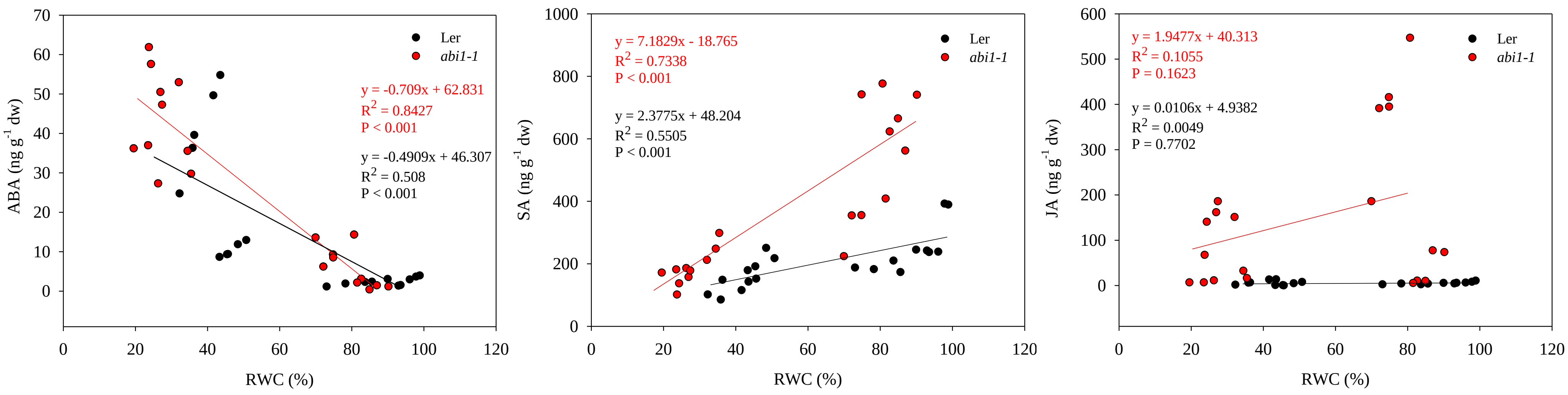

Table S1 Analysis of variance of growth characteristic parameters and hormonal concentration for *aba1-1* and *abi1-1* plants. P<0.05 denotes statistical significance.

		**P-value**		
	Parameter	Genotype	Stress	Interaction *genotype x stress*
aba1-1	FW	<0.0001	<0.0001	0.1648
	DW	<0.0001	0.0002	0.4085
	RWC	0.0008	<0.0001	0.5047
	Diameter	<0.0001	<0.0001	0.4163
	Survival	0.0085	<0.0001	0.0010
	gs	<0.0001	<0.0001	0.0007
abi1-1	FW	<0.0001	<0.0001	0.0782
	DW	<0.0001	<0.0001	0.0105
	RWC	<0.0001	<0.0001	0.0575
	Diameter	<0.0001	<0.0001	0.0005
	Survival	<0.0001	<0.0001	<0.0001
	gs	<0.0001	<0.0001	0.0250
	ABA	<0.0001	<0.0001	0.0001
	JA	<0.0001	<0.0001	<0.0001
	SA	0.0001	<0.0001	0,0086

Table S2 **Transcripts that could be under the control of *abi1-1* in response to the stress combination**

Upregulated	27
**AT number**	**Short description**
At1g52690	Late embryogenesis abundant protein (LEA) family protein; BEST Arabidopsis thaliana protein match is: Late embryogenesis abundant protein (LEA) family protein (TAIR:AT3G15670.1)
At5g06760	Late embryogenesis abundant protein LEA like (D113), encodes LEA4-5, a member of the Late Embryogenesis Abundant (LEA) proteins which typically accumulate in response to low water availability conditions imposed during development or by the environment.
At5g66400	Dehydrin RAB18-like protein, belongs to the dehydrin protein family. ABA- and drought-induced glycine-rice dehydrin protein.
At2g37870	Bifunctional inhibitor/lipid-transfer protein/seed storage 2S albumin superfamily protein.
At3g48530	SNF1-related protein kinase regulatory subunit gamma 1 (KING1); FUNCTIONS IN: molecular_function unknown; INVOLVED IN: biological_process unknown; LOCATED IN: plant-type cell wall; EXPRESSED IN: 23 plant structures; EXPRESSED DURING: 13 growth stages; CONTAINS InterPro DOMAIN/s: Cystathionine beta-synthase, core (InterPro:IPR000644)
At4g27410	Encodes a NAC transcription factor induced in response to dessication. It is localized to the nucleus and acts as a transcriptional activator in ABA-mediated dehydration response.
At1g48970	NagB/RpiA/CoA transferase-like superfamily protein; FUNCTIONS IN: GTP binding, translation initiation factor activity; INVOLVED IN: translational initiation, cellular metabolic process; LOCATED IN: eukaryotic translation initiation factor 2B complex; EXPRESSED IN: 23 plant structures; EXPRESSED DURING: 13 growth stages.
At2g39800	AtP5C1, encodes a delta1-pyrroline-5-carboxylate synthase that catalyzes the rate-limiting enzyme in the biosynthesis of proline. Expression is induced by abscisic acid and salt stress in a light-dependent manner.
At2g33380	AtRD20, encodes a calcium binding protein whose mRNA is induced upon treatment with NaCl, ABA and in response to dessication. mRNA expression under drought conditions is apparent particularly in leaves and flowers. Isoform of caleosin with a role as a peroxygenase involved in oxylipin metabolism during biotic and abiotic stress.
At4g26080	AtABI1 Involved in abscisic acid (ABA) signal transduction. Negative regulator of ABA promotion of stomatal closure.
At5g12030	heat shock protein 17.6A, encodes a cytosolic small heat shock protein with chaperone activity that is induced by heat and osmotic stress and is also expressed late in seed development.
At4g34000	abscisic acid responsive elements-binding factor (ABRE/ABF3). Encodes an ABA-responsive element-binding protein with similarity to transcription factors that is expressed in response to stress and abscisic acid.
At5g11110	Encodes a protein with putative sucrose-phosphate synthase activity.Involved in pollen exine formation.
At4g30460	glycine-rich protein; FUNCTIONS IN: molecular_function unknown; INVOLVED IN: biological_process unknown; LOCATED IN: endomembrane system; EXPRESSED IN: 20 plant structures; EXPRESSED DURING: 12 growth stages
At3g04000	ChlADR is an aldehyde reductase that catalyzes the reduction of the aldehyde carbonyl groups on saturated and alpha,beta-unsaturated aldehydes with more than 5 carbons in vitro. The N-terminal region of this protein directs GFP to the chloroplast where where ChlADR likely helps to maintain the photosynthetic process by detoxifying reactive carbonyls formed during lipid peroxidation.
At2g42540	A cold-regulated gene whose product is targeted to the chloroplast. Cor15am protects stromal proteins from aggregation under various stress conditions. Constitutive expression increases freezing tolerance in protoplasts in vitro and chloroplasts in vivo. NMR and x-ray diffraction studies suggest that COR15a alters the intrinsic curvature of the inner membrane of chloroplast envelope. Late Embryogenesis abundant protein (LEA).
At3g08860	Encodes a protein that is predicted to have beta-alanine aminotransferase activity.
At3g57540	Remorin family protein; CONTAINS InterPro DOMAIN/s: Remorin, C-terminal (InterPro:IPR005516); BEST Arabidopsis thaliana protein match is: Remorin family protein (TAIR:AT2G41870.1)
At2g47470	Encodes a protein disulfide isomerase-like (PDIL) protein, a member of a multigene family within the thioredoxin (TRX) superfamily. Transcript levels for this gene are up-regulated in response to three different chemical inducers of ER stress (dithiothreitol, beta-mercaptoethanol, and tunicamycin). AtIRE1-2 does not appear to be required for this response, but the atbzip60 mutant has a diminished response.
At3g53980	Bifunctional inhibitor/lipid-transfer protein/seed storage 2S albumin superfamily protein; FUNCTIONS IN: lipid binding; INVOLVED IN: lipid transport; LOCATED IN: endomembrane system; EXPRESSED IN: 10 plant structures; EXPRESSED DURING: 7 growth stages; CONTAINS InterPro DOMAIN/s: Bifunctional inhibitor/plant lipid transfer protein/seed storage (InterPro:IPR016140)
At5g61820	FUNCTIONS IN: molecular_function unknown; INVOLVED IN: biological_process unknown; LOCATED IN: vacuole; EXPRESSED IN: 24 plant structures; EXPRESSED DURING: 15 growth stages; CONTAINS InterPro DOMAIN/s: Stress up-regulated Nod 19 (InterPro:IPR011692)
At1g34630	BEST Arabidopsis thaliana protein match is: Mitochondrial import inner membrane translocase subunit Tim17/Tim22/Tim23 family protein (TAIR:AT5G51150.1)
At1g79520	Cation efflux family protein; FUNCTIONS IN: cation transmembrane transporter activity; INVOLVED IN: cation transport, transmembrane transport; LOCATED IN: membrane; EXPRESSED IN: 18 plant structures; EXPRESSED DURING: 10 growth stages; CONTAINS InterPro DOMAIN/s: Cation efflux protein (InterPro:IPR002524)
At3g60350	ARABIDILLO-2 and its homolog, ARABIDILLO -1, are unique among Arabidopsis Arm-repeat proteins in having an F-box motif and fall into a phylogenetically distinct subgroup from other plant Arm-repeat proteins Similar to arm repeat protein in rice and armadillo/beta-catenin repeat family protein / F-box family protein in Dictyostelium.
At2g43570	chitinase, putative (CHI); FUNCTIONS IN: chitin binding, chitinase activity; INVOLVED IN: carbohydrate metabolic process, cell wall macromolecule catabolic process; LOCATED IN: apoplast, plant-type cell wall; EXPRESSED IN: 15 plant structures; EXPRESSED DURING: 10 growth stages.
At4g16190	Papain family cysteine protease; FUNCTIONS IN: cysteine-type peptidase activity, cysteine-type endopeptidase activity; INVOLVED IN: proteolysis; LOCATED IN: vacuole; EXPRESSED IN: 23 plant structures;

At1g74310	heat shock protein 101, encodes ClpB1, which belongs to the Casein lytic proteinase/heat shock protein 100 (Clp/Hsp100) family. Involved in refolding of proteins which form aggregates under heat stress. Also known as AtHsp101. AtHsp101 is a cytosolic heat shock protein required for acclimation to high temperature.

Down regulated	23
**AT number**	**Short description**
At5g10390	Histone superfamily protein; FUNCTIONS IN: DNA binding; INVOLVED IN: nucleosome assembly; LOCATED IN: chloroplast, nucleosome; EXPRESSED IN: 23 plant structures; EXPRESSED DURING: 13 growth stages; CONTAINS InterPro DOMAIN/s: Histone H3 (InterPro:IPR000164), Histone-fold (InterPro:IPR009072), Histone core (InterPro:IPR007125)
At4g16500	Cystatin/monellin superfamily protein; FUNCTIONS IN: enzyme regulator activity, cysteine-type endopeptidase inhibitor activity; INVOLVED IN: biological_process unknown; LOCATED IN: cell wall, vacuole; EXPRESSED IN: 24 plant structures; EXPRESSED DURING: 15 growth stages.
At5g59870	Encodes HTA6, a histone H2A protein.
At2g38310	Encodes a member of the PYR (pyrabactin resistance)/PYL(PYR1-like)/RCAR (regulatory components of ABA receptor) family proteins with 14 members. PYR/PYL/RCAR family proteins function as abscisic acid sensors. Mediate ABA-dependent regulation of protein phosphatase 2Cs ABI1 and ABI2.
At4g20780	Calcium sensor involved in trichome branching.
At5g05440	Encodes a member of the PYR (pyrabactin resistance)/PYL(PYR1-like)/RCAR (regulatory components of ABA receptor) family proteins with 14 members. PYR/PYL/RCAR family proteins function as abscisic acid sensors. Mediate ABA-dependent regulation of protein phosphatase 2Cs ABI1 and ABI2.
At4g11290	peroxidase ATP19a, peroxidase superfamily protein; FUNCTIONS IN: peroxidase activity, heme binding; INVOLVED IN: response to oxidative stress, oxidation reduction; LOCATED IN: endomembrane system; EXPRESSED IN: 11 plant structures; EXPRESSED DURING: 7 growth stages.
At2g06850	endoxyloglucan transferase (EXGT-A1) gene
At2g10940	Bifunctional inhibitor/lipid-transfer protein/seed storage 2S albumin superfamily protein; FUNCTIONS IN: lipid binding; INVOLVED IN: lipid transport; LOCATED IN: chloroplast thylakoid membrane, apoplast, chloroplast, membrane; EXPRESSED IN: 21 plant structures; EXPRESSED DURING: 13 growth stages.
At2g22170	Lipase/lipooxygenase, PLAT/LH2 family protein; FUNCTIONS IN: molecular_function unknown; INVOLVED IN: biological_process unknown; LOCATED IN: thylakoid, chloroplast, membrane; EXPRESSED IN: 19 plant structures; EXPRESSED DURING: 13 growth stages.
At1g56430	Encodes a protein with nicotianamine synthase activity.
At2g14560	Encodes LURP1, a member of the LURP cluster (late upregulated in response to Hyaloperonospora parasitica) which exhibits a pronounced upregulation after recognition of the pathogenic oomycte H. parasitica. LURP1 is required for full basal defense to H. parasitica and resistance to this pathogen mediated by the R-proteins RPP4 and RPP5.
At3g05730	Encodes a defensin-like (DEFL) family protein.
At5g64290	2-oxoglutarate/malate translocator, dicarboxylate transport 2.1 (DIT2.1); FUNCTIONS IN: oxoglutarate:malate antiporter activity; INVOLVED IN: malate transport, response to nematode; LOCATED IN: chloroplast, membrane, chloroplast envelope; EXPRESSED IN: 23 plant structures; EXPRESSED DURING: 13 growth stages.
At1g67330	Protein of unknown function (DUF579); FUNCTIONS IN: molecular_function unknown; INVOLVED IN: biological_process unknown; LOCATED IN: endomembrane system; EXPRESSED IN: 8 plant structures; EXPRESSED DURING: LP.06 six leaves visible, LP.04 four leaves visible, LP.10 ten leaves visible, 4 leaf senescence stage.
At3g06750	hydroxyproline-rich glycoprotein family protein; Has 6317 Blast hits to 3436 proteins in 319 species: Archae - 0; Bacteria - 429; Metazoa - 2195; Fungi - 739; Plants - 2175; Viruses - 83; Other Eukaryotes - 696 (source: NCBI BLink).
At3g12710	DNA glycosylase superfamily protein; FUNCTIONS IN: DNA-3-methyladenine glycosylase I activity, catalytic activity; INVOLVED IN: DNA repair, base-excision repair; EXPRESSED IN: 20 plant structures; EXPRESSED DURING: 13 growth stages; CONTAINS InterPro.
At5g65360	Histone superfamily protein; FUNCTIONS IN: DNA binding; INVOLVED IN: nucleosome assembly; LOCATED IN: chloroplast, nucleosome; EXPRESSED IN: 23 plant structures; EXPRESSED DURING: 14 growth stages; CONTAINS InterPro
At5g62920	Encodes a Type-A response regulator that is responsive to cytokinin treatment. Its C-ter domain is very short in comparison to other Arabidopsis ARRs (17 total). Arr6 protein is stabilized by cytokinin.
At2g16060	class 1 non-symbiotic hemoglobin (AHB1), encodes a class 1 nonsymbiotic hemoglobin induced by low oxygen levels with very high oxygen affinity. It is not likely to be a hemoglobin transporter because of its extremely high affinity for oxygen. Overexpression impairs cold stress-induced nitric oxide (NO) production.
At1g03870	Fasciclin-like arabinogalactan-protein 9 (Fla9)
At2g44740	cyclin p4;1 (CYCP4;1); CONTAINS InterPro DOMAIN/s: Negative regulatory factor PREG (InterPro:IPR012389), Cyclin-like (InterPro:IPR011028), Cyclin, N-terminal (InterPro:IPR006671), Cyclin (InterPro:IPR006670).
At1g48630	Encodes a protein with similarity to mammalian RACKs. RACKs function to shuttle activated protein kinase C to different subcellular sites and may also function as a scaffold through physical interactions with other proteins.
